# Amygdala granular fuzzy astrocytes are independently associated with both LATE neuropathologic change and argyrophilic grains: a study of Japanese series with a low to moderate Braak stage

**DOI:** 10.1186/s40478-023-01643-5

**Published:** 2023-09-11

**Authors:** Osamu Yokota, Tomoko Miki, Hanae Nakashima-Yasuda, Hideki Ishizu, Takashi Haraguchi, Chikako Ikeda, Akinori Miyashita, Takeshi Ikeuchi, Shintaro Takenoshita, Seishi Terada, Manabu Takaki

**Affiliations:** 1 Department of Neuropsychiatry, Okayama University Graduate School of Medicine, Dentistry and Pharmaceutical Sciences, 2-5-1 Shikata-cho, Okayama, 700-8558 Japan; 2 Okayama University Medical School, Okayama, Japan; 3 Department of Psychiatry, Kinoko Espoir Hospital, Okayama, Japan; 4 Department of Neuropsychiatry, Okayama University Hospital, Okayama, Japan; 5 Department of Psychiatry, Zikei Hospital, Okayama, Japan; 6 Department of Neurology, National Hospital Organization Minami-Okayama Medical Center, Okayama, Japan; 7 Department of Molecular Genetics, Brain Research Institute, Niigata University, Niigata, Japan; 8 Department of Neuropsychiatry, Okayama University Faculty of Medicine, Dentistry and Pharmaceutical Sciences, Okayama, Japan

**Keywords:** Argyrophilic grain, ARTAG, Hippocampal sclerosis, TDP-43, PART

Limbic-predominant age-related TDP-43 encephalopathy neuropathologic change (LATE-NC) [13] frequently coexists with various neurodegenerative diseases. On the other hand, ‘pure LATE-NC’ is also drawing attention [14]. LATE-NC first develops in the amygdala.

The amygdala is also preferentially affected by argyrophilic grains (AGs) and age-related tau astrogliopathy (ARTAG). AGs are age-related lesions in which four-repeat tau is selectively accumulated [2]. AGs are readily detected by Gallyas-Braak silver stain (Gallyas method), and their distribution can be assessed by the Saito stage [18]. The formation of AGs is associated with ARTAG, especially granular fuzzy astrocytes (GFAs) which may develop prior to AGs [8, 21]. AGs and ARTAG share tau filaments with identical cryo-electron microscopy structures [19].

At present, the pathogenic relationship between LATE-NC, AGs, and GFAs in the amygdala remains to be elucidated. For example, although a few previous studies supported the potential relationship between LATE-NC and AGs [1, 4], a recent study failed to demonstrate a significant association [7], remaining controversial. Regarding the relationship between LATE-NC and ARTAG, there is only one study that demonstrated that LATE-NC was associated with the percentage of brain regions with ARTAG [3]. Further, whether these lesions have independent effects on tissue degeneration in the amygdala has not been also examined.

To address these issues, first, we examined whether amygdala GFAs and AGs are independent risk factors of LATE-NC in a Japanese series with a low to moderate Braak neurofibrillary tangle (NFT) stage. Then, independent pathological risk factors of the formation of AGs in this series were also explored. Finally, whether LATE-NC, AGs, and amygdala GFAs have independent effects on severe loss of neurons in the amygdala was examined.

From 1,180 autopsy cases who were registered in the database at the Department of Neuropsychiatry, Okayama University Graduate School of Medicine, Dentistry and Pharmaceutical Sciences as of the end of June 2023, we first selected 501 cases in which all of the following pathological data were available: Braak NFT stage, Thal phase, CERAD neuritic plaque score, LBD subtypes, Braak Parkinson’s disease stage, Saito AG stage [18], subcortical NFTs which fit the NINDS-PSP criteria, tufted astrocytes, astrocytic plaques, and GFAs in the frontal cortex and subcortical nuclei, Josephs TDP-43 stage [6], LATE-NC stage [14, 15], histological subtypes of primary TDP-43 proteinopathies, fused in sarcoma (FUS) pathology, Pick bodies, and the semiquantitative data of neuronal loss in representative anatomical regions. The staging system of GFAs (GFA stage) was as follows [10, 21]: stage 0, no lesion in the anatomical region; stage 1, more than one lesion in the anatomical region but less than one lesion per × 200 visual field; stage 2, one lesion per × 200 visual field; stage 3, two or ten lesions per × 200 visual field; or stage 4, 11 or over per × 200 visual field. Hippocampal sclerosis (HS) was defined as severe neuronal loss in the hippocampal CA1 with or without the subiculum that could not be explained by hypoxia, ischemia, or epilepsy. The degree of neuronal loss in the amygdala was semiquantitatively assessed on sections stained with hematoxylin–eosin according to a four-point staging system (none, mild, moderate, and severe) used previously (Additional file 1: Fig. S1) [20].

From 501 cases, we excluded any cases having following pathologies: NFTs with Braak stages V-VI, Lewy bodies in any regions including the olfactory bulb, NFTs which quantity fit the NINDS-PSP criteria, tufted astrocytes and astrocytic plaques in any region, amyotrophic lateral sclerosis or frontotemporal lobar degeneration with TDP-43-positive or FUS-positive inclusions, Pick’s disease, globular glial tauopathy, multiple system atrophy, trinucleotide repeat diseases, inflammatory diseases, leukodystrophies, and lysosomal storage diseases. Finally, 72 cases that were Braak NFT stages I-IV and Thal phases 0–4 but lacked any other neurodegenerative changes except for AGs, GFAs, LATE-NC, or HS which is closely associated with LATE-NC, were extracted (Table 1, Additional file 2: Table S1). Details of pathological examination and statistical analysis were shown in (Additional file 3: File S1).

**Table 1:**
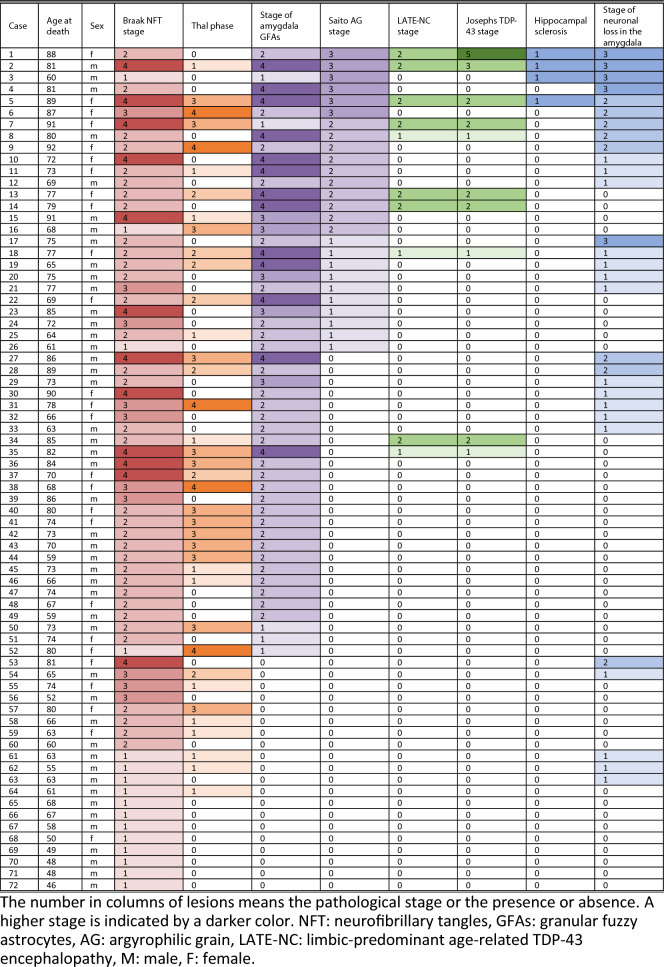
All 72 cases with Braak NFT stage I to IV

The mean age at death in 72 cases was 71.6 ± 11.6 years. Of 72 cases, 52 cases had GFAs in the amygdala (72.2%), and 26 had AGs (36.1%) (Table 1, Fig. 1C and D, Additional file 1: Fig. S1). All AG-positive cases had amygdala GFAs (Table 1). LATE-NC was noted in 10 of 72 cases (13.9%, Table 1, Fig. 1A and B, Additional file 4: Fig. S2). All LATE-NC cases also had amygdala GFAs (Table 1). The LATE-NC stage was significantly correlated with the amygdala GFA stage (*ρ* = 0.3969, *p* < 0.001, Fig. 1G. Spearman’s rank-order correlation test). Likewise, the LATE-NC stage was significantly correlated with Saito AG stage (*ρ* = 0.4324,* p* < 0.001, Spearman’s rank-order correlation test).

**Fig. 1:**
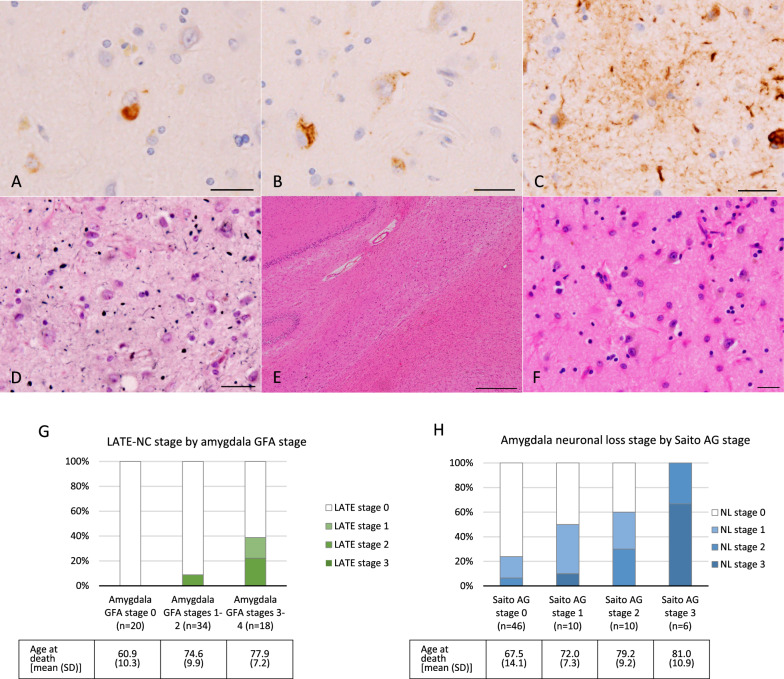
Phosphorylated TDP-43 pathology, amygdala GFAs, AGs, HS, and severe amygdala degeneration. **A**–**F** Pathological findings in a case with Braak NFT stage IV, Thal phase 1, amygdala GFA stage 4, Saito AG stage 3, LATE-NC stage 2. **A**, **B** Phosphorylated TDP-43 accumulation in the amygdala. ps409/410 immunohistochemistry. Scale bar: 25 μm. **C** A GFA in the amygdala. AT8 immunohistochemistry. Scale bar: 25 μm. **D** Argyrophilic grains in the amygdala. Gallyas method. Counterstaining with hematoxylin–eosin stain. Scale bar: 25 μm. **E** HS showing severe loss of pyramidal neurons in the hippocampal CA1. Hematoxylin–eosin stain. Scale bar: 500 μm. **F** Severe loss of neurons with gliosis in the amygdala. Hematoxylin–eosin stain. Scale bar: 25 μm. **G** The frequency of LATE-NC-positive cases by amygdala GFA stage. All LATE-NC-positive cases were amygdala GFA-positive, and the frequency of LATE-NC gradually increased with amygdala GFA stage (0% in GFA stage 0, 8.8% in GFA stages 1–2, 38.9% in GFA stages 3–4). The age at death [mean (standard deviation)] in each group is also shown. **H** The severity of neuronal loss in the amygdala by Saito AG stage. The age at death [mean (standard deviation)] is also shown.

Four of 72 cases (5.6%) had HS (Figs. 1E). All HS cases had amygdala GFAs and AGs, while only three HS cases had LATE-NC (Fig. 1F, Table 1). HS was noted only in cases bearing AGs with Saito stage III.

Among 72 cases, five (6.9%) had severe neuronal loss in the amygdala (Table 2). Of the five cases, all cases had amygdala GFAs, four had AGs with Saito stage III, and two had LATE-NC. Saito AG stage was significantly correlated with the severity of neuronal loss in the amygdala (ρ = 0.5043, *p* < 0.001, Fig. 1H. Spearman’s rank-order correlation test).

**Table 2:** Univariate and multivariate binomial logistic regression analyses of risk factors associated with LATE-NC, argyrophilic grains, and severe neuronal loss in the amygdala

	Univariate analysis		Multivariate analysis	
	Odds ratio (95% CI)	*P* value	Odds ratio (95% CI)	*P* value
*LATE-NC*				
Age at death	1.16 (1.05–1.27)	0.0029**	1.27 (1.03–1.56)	0.0264*
Sex (female)	3.15 (0.80–12.43)	0.1013	Excluded	–
Braak NFT stages IV	4.50 (1.04–19.51)	0.0445*	0.44 (0.05–3.90)	0.4616
Presence of Aβ deposits	2.33 (0.55–9.86)	0.2492	Excluded	–
Thal phases 3–4	1.34 (0.31–5.85)	0.6947	Excluded	–
Amygdala GFA stage 4	21.78 (4.43–107.13)	< 0.001**	40.7 (2.81–590.25)	0.0066**
Presence of AGs	9.78 (1.89–50.59)	0.0066**	Excluded	–
Saito AG stages II–III	13.74 (2.99–63.20)	< 0.001**	2.02 (0.27–14.88)	0.4915
Infarctions in the neocortex	0.58 (0.07–5.16)	0.6255	Excluded	-
Infarctions in the subcortical nuclei	0.90 (0.21–3.91)	0.8934	Excluded	–
*Argyrophilic grains*				
Age at death	1.07 (1.02–1.13)	0.0058**	1.07 (1.00–1.14)	0.0428*
Sex (female)	2.03 (0.64–6.44)	0.2283	Excluded	–
Braak NFT stage IV	2.00 (0.57–7.00)	0.2780	0.39 (0.07–2.24)	0.2905
Presence of Aβ deposits	0.84 (0.32–2.20)	0.7227	Excluded	–
Thal phase 4	1.19 (0.18–7.65)	0.8513	Excluded	–
Amygdala GFA stage 4	16.13 (3.20–81.25)	< 0.001**	12.43 (1.97–78.37)	0.0073**
Presence of LATE-NC	9.78 (1.89–50.59)	0.0066**	1.70 (0.22–13.26)	0.6111
Infarctions in the neocortex	2.11 (0.54–8.23)	0.2817	Excluded	–
Infarctions in the subcortical nuclei	0.91 (0.30–2.70)	0.8599	Excluded	–
*Severe neuronal loss in the amygdala*				
Age at death	1.05 (0.96–1.15)	0.2858	0.98 (0.88–1.10)	0.7429
Sex (female)	0.42 (0.04–3.97)	0.4491	Excluded	–
Braak NFT stage IV	1.27 (0.13–12.50)	0.8361	Excluded	–
Presence of Aβ deposits	0.20 (0.02–1.91)	0.1632	Excluded	–
Amygdala GFA stage 4	3.39 (0.51–22.75)	0.2080	0.73 (0.06–8.24)	0.7978
Saito AG stage I	1.61 (0.16–16.09)	0.6846	Excluded	–
Saito AG stages II–III	18.33 (1.88–178.98)	0.0123*	21.00 (1.34–328.20)	0.0300*
Saito AG stage III	130.00 (9.62–1757.58)	< 0.001**	Excluded	–
Presence of LATE-NC	4.92 (0.71–34.06)	0.1068	1.67 (0.12–23.23)	0.7026
Infarctions in the neocortex	1.96 (0.18–21.02)	0.5772	Excluded	–
Infarctions in the subcortical nuclei	0.70 (0.07–7.16)	0.7637	Excluded	–

*N* Number of cases, *LATE-NC* Limbic-predominant age-related TDP-43 encephalopathy neuropathologic changes, *NFT* Neurofibrillary tangle, *GFA* Granular fuzzy astrocyte, *AG* Argyrophilic grain, *CI* Confidence interval

*: *p* < 0.05, **: *p* < 0.01. Severe neuronal loss in the amygdala means stage 3 according to the staging system of neuronal loss applied in the present study

Multivariate binomial logistic regression analysis (independent variables: the age at death, Braak NFT stage IV, amygdala GFA stage 4, and Saito AG stages II–III) demonstrated that the age at death (odds ratio [95% confidence interval] 1.27 [1.03–1.56]; *p* = 0.0264) and amygdala GFA stage 4 (40.7 [2.81–590.25]; *p* = 0.0066) were significant predictors of LATE-NC (Table 2).

Likewise, multivariate binomial logistic regression (independent variables: the age at death, Braak NFT stage IV, amygdala GFA stage 4, and presence of LATE-NC) revealed that the age at death (1.07 [1.00–1.14]; *p* = 0.0428) and amygdala GFA stage 4 (12.43 [1.97–78.37], *p* = 0.0073) were independent risk factors of AGs (Table 2).

In the multivariate binomial logistic regression regarding tissue degeneration in the amygdala (independent variables: the age at death, amygdala GFA stage 4, Saito AG stages II-III, and presence of LATE-NC), only Saito AG stages II-III (21.00 [1.34–328.20], *p* = 0.0300) was an independent risk factor of severe neuronal loss with stage 3.

These findings suggest that amygdala GFAs, but not AGs, may be a potential factor associated with the occurrence of LATE-NC in cases with Braak NFT stages I-IV. In the relationship between GFAs and LATE-NC, a recent study demonstrated that LATE-NC was associated with the percentage of brain regions with ARTAG [3]. Although the effect of GFAs in the amygdala was not separately examined in the study, it was consistent with our findings in terms of pointing out the potential relationship between GFAs and LATE-NC. On the other hand, previous findings regarding the relationship between AGs and LATE-NC are not consistent. For example, while it was reported that the frequency of AGs in LATE-NC-positive cases was significantly higher than that in LATE-NC-negative cases [1], a recent study failed to demonstrate their association [7]. In these previous studies, the effect of amygdala GFAs was not considered in statistical analyses. The inconsistency in the results might be partially explained by this difference in the methodological setting between studies. On the other hand, the possibility that AGs have some effect on the formation of LATE-NC as a confounder cannot be denied, and the possible effect should be examined using multivariate analysis in which the effect of amygdala GFAs is considered and a larger number of cases are employed. Furthermore, given our findings, amygdala GFAs may be common pathological factors that are involved in the formation process of LATE-NC and in that of AGs. Indeed, in our series, LATE-NC and AGs were observed only in cases bearing amygdala GFAs. The possibility that LATE-NC and AGs may share some pathological pathway involving amygdala GFAs should be further examined.

Finally, in our series, multivariate analysis demonstrated that AGs with Saito stages II-III (i.e., AGs that extend from the limbic system to the temporal cortex) independently contribute to amygdala degeneration. Although LATE-NC was not a statistically significant factor in the present study, it might be explained by the small number of LATE-NC-positive cases in our series. Indeed, it was reported that LATE-NC was associated with the amygdala volume assessed using postmortem MRI [9]. Regarding the influence of AGs on cognitive functions, an early study demonstrated that cases bearing AGs with Saito stage III (AGs that extend to the insular cortex and gyrus rectus) almost consistently showed cognitive impairment [18]. However, because subsequent studies in which the presence or absence of AGs or AGs only in the limbic region were assessed did not demonstrate the significant association [5, 12, 16, 17], clinical impact of AGs is questioned. On the other hand, there are also several studies that revealed the statistically significantly higher frequencies of appetite/eating abnormalities [16], suicide [22], and late-onset psychotic disorder [11] in cases bearing AGs compared with cases lacking them. Taking these findings, together with our results that AGs can contribute to amygdala degeneration, into consideration, the clinical influence of AGs may need to be reexamined by focusing on Saito stages II-III in which AGs extend to the temporo-frontal cortex.

### Supplementary Information

**Additional file 1: Fig. S1.** Representative figures of neuronal loss stage in the amygdala. **A**–**C** Stage 0. Neuronal loss and glial proliferation is absent. **D**–**F** Stage 1. Mild neuronal loss with minimal gliosis is noted. **G**–**I** Stage 2. Moderate neuronal loss with gliosis is present, but tissue rarefaction is not evident. **J**–**L** Stage 3. Severe neuronal loss with remarkable glial proliferation is noted. Tissue rarefaction is also seen. Scale bars: **A**, **D**, **G**, **J** 200 μm, **B**, **E**, **H**, **K** 100 μm, **C**, **F**, **I**, **L** 50 μm.

**Additional file 2: Table S1.** Demographic data in cases with Braak stages I-IV by LATE-NC status.

**Additional file 3: File S1.** Supplementary file 1: Supplementary materials and methods

**Additional file 4: Fig. S2.** TDP-43 pathology, granular fuzzy astrocytes (GFAs), argyrophilic grains, hippocampal sclerosis, and tissue degeneration in the amygdala in representative cases. **A**–**F** Pathological findings in a case with Braak NFT stage II, Thal phase 0, amygdala GFA stage 2, Saito AG stage III, and LATE-NC stage 2. **A**, **B** Phosphorylated TDP-43 accumulation in the amygdala **A** and dentate gyrus in the hippocampus **B**. pS409/410 immunohistochemistry. Scale bar: 25 μm. **C** A GFA in the amygdala. AT8 immunohistochemistry. Scale bar: 25 μm. **D** AGs in the amygdala. Gallyas method. Scale bar: 25 μm. **E** Hippocampal sclerosis. Hematoxylin-eosin stain. Scale bar: 100 μm. **F** Severe loss of neurons with gliosis in the amygdala. Hematoxylin-eosin stain. Scale bar: 25 μm. **G**–**L** Pathological findings in a case with Braak stage II, Thal phase 0, amygdala GFA stage 4, Saito AG stage III, and LATE-NC stage 0. **G** This case lacked phosphorylated TDP-43-positive lesion in any region. The amygdala. pS409/410 immunohistochemistry. Scale bar: 25 μm. **H**, **I** GFAs in the amygdala. AT8 immunohistochemistry. Scale bar: 25 μm. **J** AGs in the amygdala. Gallyas method. Scale bar: 25 μm. **K** Neither loss of pyramidal neurons nor gliosis is noted in the hippocampal CA1. Hematoxylin-eosin stain. Scale bar: 100 μm. **L** Severe neuronal loss with gliosis in the amygdala. Hematoxylin-eosin stain. Scale bar: 25 μm.

## Data Availability

All data of this study are provided in the Tables and Supplementary Table.

## Abbreviations

LATE-NC: Limbic-predominant age-related TDP-43 encephalopathy neuropathologic change
AG: Argyrophilic grain
ARTAG: Age-related tau astrogliopathy
GFA: Granular fuzzy astrocyte
NFT: Neurofibrillary tangle
FUS: Fused in sarcoma
HS: Hippocampal sclerosis
