# Supplementary material for: Amygdala granular fuzzy astrocytes are independently associated with both LATE neuropathologic change and argyrophilic grains: a study of Japanese series with a low to moderate Braak stage
Source: Acta Neuropathol Commun. 2023 Sep 11;11:148. doi: 10.1186/s40478-023-01643-5 (PMC10496338; doi:10.1186/s40478-023-01643-5)
Supplement: Supplementary file 1 — Additional file 1: Fig. S1. Representative figures of neuronal loss stage in the amygdala. A–C Stage 0. Neuronal loss and glial proliferation is absent. D–F Stage 1. Mild neuronal loss with minimal gliosis is noted. G–I Stage 2. Moderate neuronal loss with gliosis is present, but tissue rarefaction is not evident. J–L Stage 3. Severe neuronal loss with remarkable glial proliferation is noted. Tissue rarefaction is also seen. Scale bars: A, D, G, J 200 μm, B, E, H, K 100 μm, C, F, I, L 50 μm. [file 40478_2023_1643_MOESM1_ESM.pptx]

## Slide 1
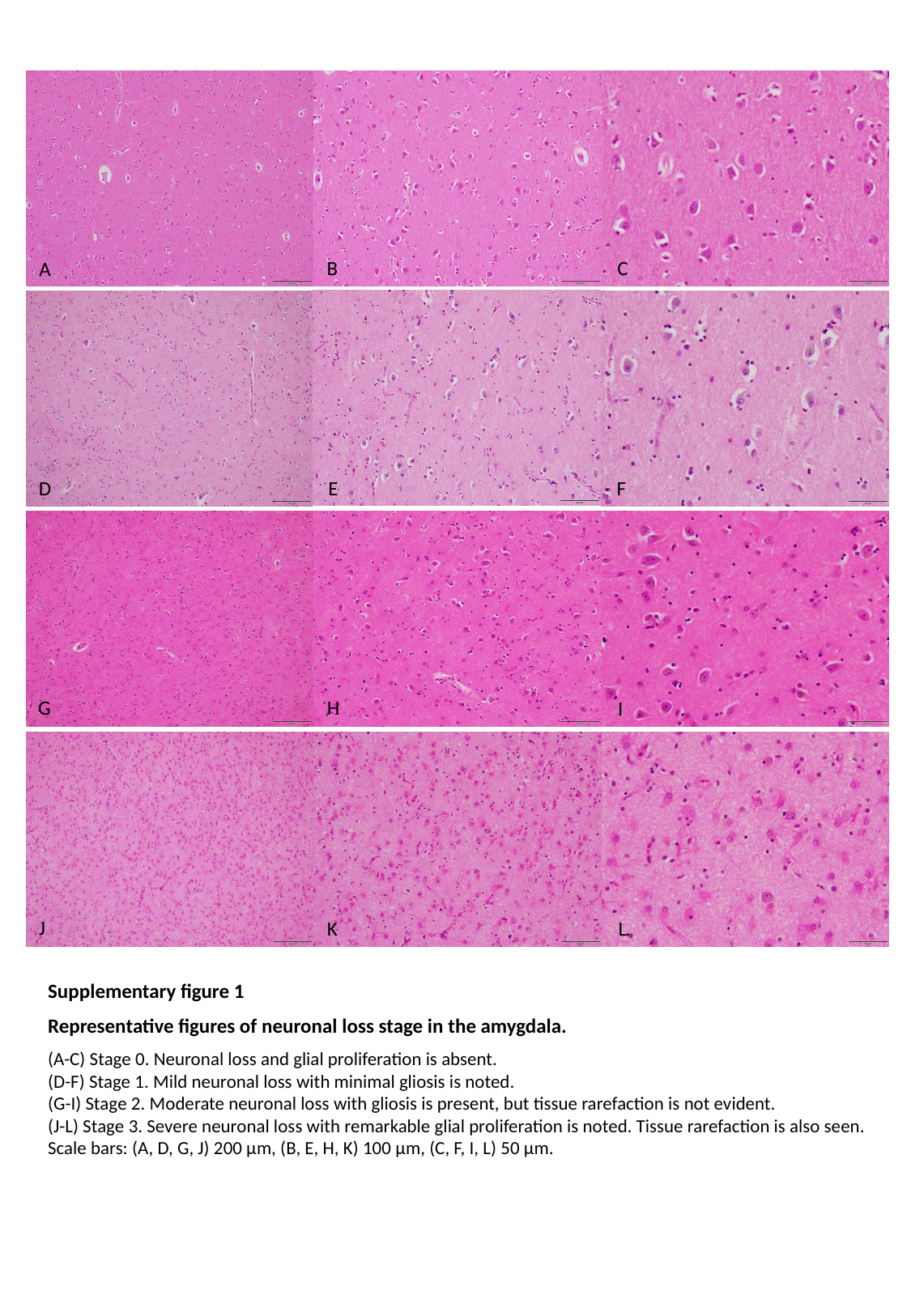

C
B
A
F
E
D
G
H
I
J
L
K
Supplementary figure 1
Representative figures of neuronal loss stage in the amygdala.
(A-C) Stage 0. Neuronal loss and glial proliferation is absent.
(D-F) Stage 1. Mild neuronal loss with minimal gliosis is noted.
(G-I) Stage 2. Moderate neuronal loss with gliosis is present, but tissue rarefaction is not evident.
(J-L) Stage 3. Severe neuronal loss with remarkable glial proliferation is noted. Tissue rarefaction is also seen.
Scale bars: (A, D, G, J) 200 μm, (B, E, H, K) 100 μm, (C, F, I, L) 50 μm.
