# Supplementary material for: Amygdala granular fuzzy astrocytes are independently associated with both LATE neuropathologic change and argyrophilic grains: a study of Japanese series with a low to moderate Braak stage
Source: Acta Neuropathol Commun. 2023 Sep 11;11:148. doi: 10.1186/s40478-023-01643-5 (PMC10496338; doi:10.1186/s40478-023-01643-5)
Supplement: Supplementary file 2 — Additional file 2: Table S1. Demographic data in cases with Braak stages I-IV by LATE-NC status. [file 40478_2023_1643_MOESM2_ESM.docx]

**Supplementary Table 1**

**Demographic data in cases with Braak stages I-IV by LATE-NC status**

|  | Cases with LATE-NC | Cases without LATE-NC | *P* value |
| --- | --- | --- | --- |
| N (%) | 10 (13.9) | 62 (86.1) |  |
| Sex (female) | 6 (60.0) | 20 (32.3) | 0.1530 |
| Age at death (y, mean ± SD) | 82.9 ± 5.1 | 69.8 ± 11.3 | 0.000185** |
| Josephs TDP-43 stage |  |  |  |
| Stage 6 | 0 (0.0) | 0 (0.0) |  |
| Stage 5 | 1 (10.0) | 0 (0.0) |  |
| Stage 4 | 0 (0.0) | 0 (0.0) |  |
| Stage 3 | 1 (10.0) | 0 (0.0) |  |
| Stage 2 | 5 (50.0) | 0 (0.0) |  |
| Stage 1 | 3 (30.0) | 0 (0.0) |  |
| Stage 0 | 0 (0.0) | 0 (0.0) |  |
| Median (25 percentile, 75 percentile) | 2.0 (1.25, 2.0) | 0 (0.0, 0.0) |  |
| LATE-NC stage |  |  |  |
| Stage 3 (N, %) | 0 (0.0) | 0 (0.0) |  |
| Stage 2 (N, %) | 7 (70.0) | 0 (0.0) |  |
| Stage 1 (N, %) | 3 (30.0) | 0 (0.0) |  |
| Stage 0 (N, %) | 0 (0.0) | 62 (100.0) |  |
| Median (25 percentile, 75 percentile) | 2.0 (1.25, 2.0) | 0 (0.0, 0.0) |  |
| Braak NFT stage |  |  |  |
| Stage VI (N, %) | 0 (0.0) | 0 (0.0) |  |
| Stage V (N, %) | 0 (0.0) | 0 (0.0) |  |
| Stage IV (N, %) | 4 (40.0) | 8 (12.9) |  |
| Stage III (N, %) | 0 (0.0) | 10 (16.1) |  |
| Stage II (N, %) | 6 (60.0) | 28 (45.2) |  |
| Stage I (N, %) | 0 (0.0) | 16 (25.8) |  |
| Stage 0 (N, %) | 0 (0.0) | 0 (0.0) |  |
| Median (25 percentile, 75 percentile) | 2 (2.0, 4.0) | 1 (1.25, 3.0) | 0.0773 |
| Thal phase |  |  |  |
| Phase 5 (N, %) | 0 (0.0) | 0 (0.0) |  |
| Phase 4 (N, %) | 0 (0.0) | 5 (8.1) |  |
| Phase 3 (N, %) | 3 (30.0) | 10 (16.1) |  |
| Phase 2 (N, %) | 2 (20.0) | 5 (8.1) |  |
| Phase 1 (N, %) | 2 (20.0) | 11 (17.7) |  |
| Phase 0 (N, %) | 3 (30.0) | 31 (50.0) |  |
| Median (25 percentile, 75 percentile) | 1.5 (0.25, 2.75) | 0.5 (0, 2.0) | 0.3545 |
| Amygdala GFA stage |  |  |  |
| Positive cases (N, %) | 10 (100.0) | 42 (67.7) | 0.0526 |
| Stage 4 (N, %) | 7 (70.0) | 6 (9.7) |  |
| Stage 3 (N, %) | 0 (0.0) | 5 (8.1) |  |
| Stage 2 (N, %) | 2 (20.0) | 27 (43.5) |  |
| Stage 1 (N, %) | 1 (10.0) | 4 (6.5) |  |
| Stage 0 (N, %) | 0 (0.0) | 20 (32.3) |  |
| Median (25 percentile, 75 percentile) | 4.0 (2.5, 4.0) | 2 (0, 2.0) | 0.000323** |
| Saito AG stage |  |  |  |
| Positive cases (N, %) | 8 (80.0) | 18 (29.0) | 0.0033** |
| Stage III (N, %) | 3 (30.0) | 3 (4.8) |  |
| Stage II (N, %) | 4 (40.0) | 6 (9.7) |  |
| Stage I (N, %) | 1 (10.0) | 9 (14.5) |  |
| Stage 0 (N, %) | 2 (20.0) | 44 (71.0) |  |
| Median (25 percentile, 75 percentile) | 2.0 (1.25, 2.75) | 0 (0, 1) | 0.000153** |
| HS (N, %) | 3 (30.0) | 1 (1.6) | 0.0074** |
| Infarctions |  |  |  |
| Neocortex | 1 (10.0) | 9 (16.1) | 0.6970 |
| Subcortical nuclei | 3 (30.0) | 18 (32.1) | 1.0000 |

N: Number of cases, y: years, SD: standard deviation, LATE-NC: limbic-predominant age-related TDP-43 encephalopathy neuropathologic changes, NFT: neurofibrillary tangle, GFA: granular fuzzy astrocyte, AGD: argyrophilic grain disease, HS: hippocampal sclerosis, FTLD-TDP: frontotemporal lobar degeneration with TDP-43-positive inclusions, ALS-TDP: amyotrophic lateral sclerosis with TDP-43-positive inclusions, FLD-FUS, frontotemporal lobar degeneration with fused in sarcoma-positive inclusions, *: *p* < 0.05, **: *p* < 0.01. The age at death, amygdala GFA stage, Saito AGD stage, and the frequency of HS in cases with LATE-NC were significantly higher than those without it (p < 0.01, respectively. Mann-Whitney U test or Fisher’s exact test).
