# Supplementary material for: Amygdala granular fuzzy astrocytes are independently associated with both LATE neuropathologic change and argyrophilic grains: a study of Japanese series with a low to moderate Braak stage
Source: Acta Neuropathol Commun. 2023 Sep 11;11:148. doi: 10.1186/s40478-023-01643-5 (PMC10496338; doi:10.1186/s40478-023-01643-5)
Supplement: Supplementary file 3 — Additional file 3: File S1. Supplementary file 1: Supplementary materials and methods [file 40478_2023_1643_MOESM3_ESM.docx]

**Supplementary file 1**

**Materials and methods**

**Conventional neuropathological assessment and immunohistochemistry**

Brain tissue samples were fixed post mortem with 10% formaldehyde and embedded in paraffin. Ten-μm-thick sections from the frontal, temporal, parietal, occipital, insular, and cingulate cortices, hippocampus, amygdala, basal ganglia, midbrain, pons, medulla oblongata, and cerebellum were prepared. These sections were stained with hematoxylin-eosin (H&E), Klüver-Barrera, Gallyas-Braak silver, and modified Bielschowsky silver methods.

Paraffin sections were immunostained by the immunoperoxidase method using 3, 3’-diaminobenzidine tetrahydrochloride. Six-μm-thick paraffin sections were immunostained by the immunoperoxidase method using 3, 3’-diaminobenzidine tetrahydrochloride. Antibodies used were against tau phosphorylated at Ser 202 (mouse, monoclonal, clone AT8; Innogenetics, Ghent, Belgium; 1:1000), 3-repeat (3R) tau (RD3, mouse, monoclonal, clone 8E6/C11; Millipore, Temecula, CA, USA; 1:2000), 4R tau (anti-4R tau, rabbit, polyclonal, Catalog No. TIP-4RT-P01; Cosmo Bio Co., Tokyo, Japan; 1:2000), phosphorylated TDP-43 (pS409/410-2, rabbit, polyclonal, Catalog No. TIP-PTD-P02; Cosmo Bio Co., Tokyo, Japan; 1:5000), FUS (rabbit, polyclonal; Catalog No. HPA008784; Sigma-Aldrich, St. Louis, MO, USA; 1:200), phosphorylated neurofilament (SMI31, mouse, monoclonal; Sternberger, Lutherville, MD, USA; 1:1000), N-terminus of p62 protein (p62-N, guinea pig, polyclonal; Progen, Heidelberg, Germany; 1:100), C-terminus of p62 protein (p62-C, guinea pig, polyclonal; Progen Biotechnik GmbH; 1:500), Aβ (11-28) (mouse, monoclonal, clone 12B2; IBL, Fujioka, Japan; 1:100), and phosphorylated α-synuclein (mouse, monoclonal, clone pSyn#64; Wako Co. Ltd., Osaka, Japan; 1:5000).

Deparaffinized sections were incubated with 1% H_2_O_2_ in methanol for 20 min to eliminate endogenous peroxidase activity in the tissue. Sections were treated with 0.2% TritonX-100 for 5 min and washed in phosphate-buffered saline (PBS, pH 7.4). After blocking with 10% normal serum, sections were incubated overnight at 4°C with one of the primary antibodies in 0.05 M Tris-HCl buffer, pH 7.2, containing 0.1% Tween and 15 mM NaN_3_. After three 10-min washes in PBS, sections were incubated in biotinylated anti-rabbit, -mouse, or -pig secondary antibody for 1 h, and then in avidin-biotinylated horseradish peroxidase complex (ABC Elite kit, Vector) for 1 h. The peroxidase labeling was visualized with diaminobenzidine as the chromogen.

**Pathological diagnoses**

Alzheimer disease (AD) (Gallyas method, AT8, 12B2, modified Bielschowsky silver stain) [2,11,12,18], primary age-related tauopathy (PART) (AT8) [4], Lewy body disease (psyn#64) [11], argyrophilic grains (Gallyas method, AT8, 4R tau) [17], PSP (Gallyas method, AT8, 4R tau) [6], CBD (Gallyas method, AT8, 4R tau) [5], Pick’s disease (AT8, RD3, modified Bielschowsky silver stain) [13,19], frontotemporal lobar degeneration with TDP-43-positive inclusions (ps409/410) [3,8,9], limbic-predominant age-related TDP-43 encephalopathy pathologies (ps409/410) [14,15,16], globular glial tauopathy (Gallyas method, AT8, 4R tau) [1], and GFAs (AT8) [7] were evaluated in all subjects using established criteria, respectively.

**Statistical analysis**

The Mann-Whitney U test and Fisher’s exact test were used to compare the variables between two groups. Spearman rank order correlation analysis was applied for univariate correlations between two variables. To assess the effects of predictor variables on the formation of LATE-NC, the formation of AGs, and severe loss of neurons in the amygdala, we constructed univariate and multivariate binomial logistic regression models, respectively. The data regarding LATE-NC status (presence or absence), AG status (presence or absence), and severe neuronal loss in the amygdala (neuronal loss stage 3 or not) were submitted as the dependent variable in the models, respectively. Independent variables in univariate and multivariate binomial regression analyses are shown in Table 2. Odds ratios (ORs) and 95% confidence intervals (CIs) were calculated after controlling simultaneously for potential confounders. A P value <0.05 was accepted as significant. Statistical analysis was performed using BellCurve for Excel 2.15 (Social Survey Research Information Co., Ltd., Tokyo, Japan).

**References**

1. Ahmed Z, Bigio EH, Budka H, Dickson DW, Ferrer I, Ghetti B et al. (2013) Globular glial tauopathies (GGT): consensus recommendations. Acta Neuropathol 126:537–544
2. Braak H, Alafuzoff I, Arzberger T, Kretzschmar H, Del Tredici K (2006) Staging of Alzheimer disease-associated neurofibrillary pathology using paraffin sections and immunocytochemistry. Acta Neuropathol 112:389-404
3. Cairns NJ, Bigio EH, Mackenzie IR, Neumann M, Lee VM, Hatanpaa KJ et al. (2007) Neuropathologic diagnostic and nosologic criteria for frontotemporal lobar degeneration: consensus of the Consortium for Frontotemporal Lobar Degeneration. Acta Neuropathol 114:5-22
4. Crary JF, Trojanowski JQ, Schneider JA, Abisambra JF, Abner EL, Alafuzoff I et al. (2014) Primary age-related tauopathy (PART): a common pathology associated with human aging. Acta Neuropathol 128:755-766
5. Dickson DW, Bergeron C, Chin SS, Duyckaerts C, Horoupian D, Ikeda K et al. (2002) Office of Rare Diseases neuropathologic criteria for corticobasal degeneration. J Neuropathol Exp Neurol 61:935–946
6. Hauw JJ, Daniel SE, Dickson D, Horoupian DS, Jellinger K, Lantos PL et al. (1994) Preliminary NINDS neuropathologic criteria for Steele-Richardson-Olszewski syndrome (progressive supranuclear palsy). Neurology 44:2015-2019
7. Kovacs GG, Ferrer I, Grinberg LT, Alafuzoff I, Attems J, Budka H et al. (2016) Aging-related tau astrogliopathy (ARTAG): harmonized evaluation strategy. Acta Neuropathol 131:87-102
8. Lee EB, Porta S, Michael Baer G, Xu Y, Suh E, Kwong LK et al. (2017) Expansion of the classification of FTLD-TDP: distinct pathology associated with rapidly progressive frontotemporal degeneration. Acta Neuropathol 134:65-78
9. Mackenzie IR, Neumann M, Baborie A, Sampathu DM, Du Plessis D, Jaros E et al. (2011) A harmonized classification system for FTLD-TDP pathology. Acta Neuropathol 122:111-113
10. McKeith IG, Dickson DW, Lowe J, Emre M, O’Brien JT, Feldman H et al. (2005) Diagnosis and management of dementia with Lewy bodies: third report of the DLB Consortium. Neurology 65:1863-1872
11. Mirra SS, Heyman A, McKeel D, Sumi SM, Crain BJ, Brownlee LM et al. (1991) The Consortium to Establish a Registry for Alzheimer’s Disease (CERAD). Part II. Standardization of the neuropathologic assessment of Alzheimer’s disease. Neurology 41:479-486
12. Montine TJ, Phelps CH, Beach TG, Bigio EH, Cairns NJ, Dickson DW et al. (2012) National Institute on Aging-Alzheimer's Association guidelines for the neuropathologic assessment of Alzheimer's disease: a practical approach. Acta Neuropathol 123:1-11
13. Munoz DG, Morris HR, Rossor M (2001) Pick’s disease. In: Neurodegeneration: The Molecular Pathology of Dementia and Movement Disorders, Second Edition, Dickson DW, Weller RO, editors. Chapter 16, pp. 156–164, Wiley-Blackwell Press: Oxford.
14. Nelson PT, Dickson DW, Trojanowski JQ, Jack CR, Boyle PA, Arfanakis K (2019) Limbic-predominant age-related TDP-43 encephalopathy (LATE): consensus working group report. Brain 142:1503-1527
15. Nelson PT (2021) LATE neuropathologic changes with little or no Alzheimer disease is common and is associated with cognitive impairment but not frontotemporal dementia. J Neuropathol Exp Neurol 80:649-651
16. Nelson PT, Lee EB, Cykowski MD, Alafuzoff I, Arfanakis K, Attems J (2023) LATE-NC staging in routine neuropathologic diagnosis: an update. Acta Neuropathol 145:159-173
17. Saito Y, Ruberu NN, Sawabe M, Arai T, Tanaka N, Kakuta Y et al. (2004) Staging of argyrophilic grains: an age-associated tauopathy. J Neuropathol Exp Neurol 63:911-918
18. Thal DR, Rüb U, Orantes M, Braak H (2002) Phases of A beta-deposition in the human brain and its relevance for the development of AD. Neurology 58:1791-1800
19. Yokota O, Tsuchiya K, Arai T, Yagishita S, Matsubara O, Mochizuki A et al. (2009) Clinicopathological characterization of Pick's disease versus frontotemporal lobar degeneration with ubiquitin/TDP-43-positive inclusions. Acta Neuropathol 117:429-444
