# Supplementary material for: Amygdala granular fuzzy astrocytes are independently associated with both LATE neuropathologic change and argyrophilic grains: a study of Japanese series with a low to moderate Braak stage
Source: Acta Neuropathol Commun. 2023 Sep 11;11:148. doi: 10.1186/s40478-023-01643-5 (PMC10496338; doi:10.1186/s40478-023-01643-5)
Supplement: Supplementary file 4 — Additional file 4: Fig. S2. TDP-43 pathology, granular fuzzy astrocytes (GFAs), argyrophilic grains, hippocampal sclerosis, and tissue degeneration in the amygdala in representative cases. A–F Pathological findings in a case with Braak NFT stage II, Thal phase 0, amygdala GFA stage 2, Saito AG stage III, and LATE-NC stage 2. A, B Phosphorylated TDP-43 accumulation in the amygdala A and dentate gyrus in the hippocampus B. pS409/410 immunohistochemistry. Scale bar: 25 μm. C A GFA in the amygdala. AT8 immunohistochemistry. Scale bar: 25 μm. D AGs in the amygdala. Gallyas method. Scale bar: 25 μm. E Hippocampal sclerosis. Hematoxylin-eosin stain. Scale bar: 100 μm. F Severe loss of neurons with gliosis in the amygdala. Hematoxylin-eosin stain. Scale bar: 25 μm. G–L Pathological findings in a case with Braak stage II, Thal phase 0, amygdala GFA stage 4, Saito AG stage III, and LATE-NC stage 0. G This case lacked phosphorylated TDP-43-positive lesion in any region. The amygdala. pS409/410 immunohistochemistry. Scale bar: 25 μm. H, I GFAs in the amygdala. AT8 immunohistochemistry. Scale bar: 25 μm. J AGs in the amygdala. Gallyas method. Scale bar: 25 μm. K Neither loss of pyramidal neurons nor gliosis is noted in the hippocampal CA1. Hematoxylin-eosin stain. Scale bar: 100 μm. L Severe neuronal loss with gliosis in the amygdala. Hematoxylin-eosin stain. Scale bar: 25 μm. [file 40478_2023_1643_MOESM4_ESM.pptx]

## Slide 1
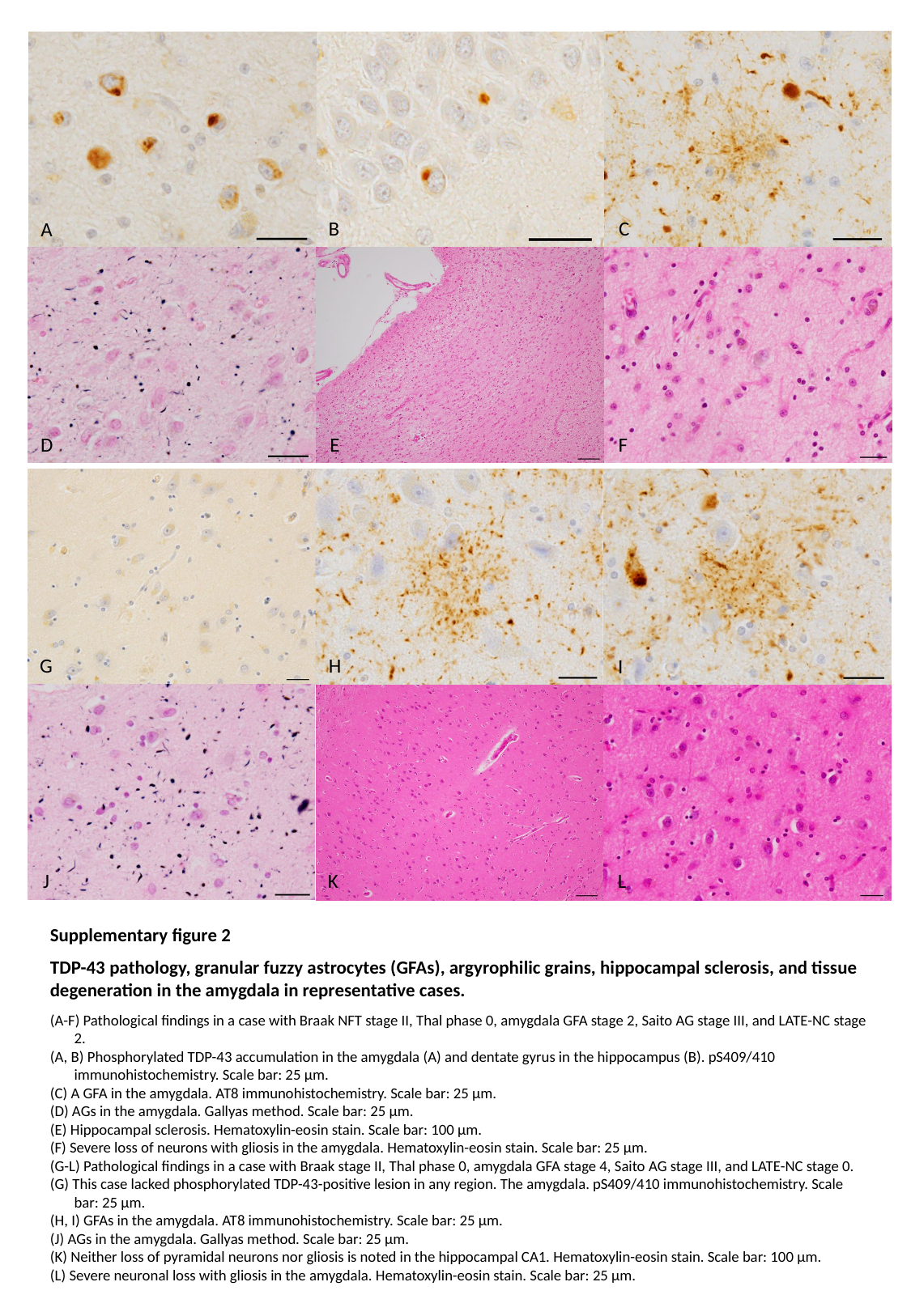

C
B
A
F
E
D
G
H
I
L
J
K
Supplementary figure 2
TDP-43 pathology, granular fuzzy astrocytes (GFAs), argyrophilic grains, hippocampal sclerosis, and tissue degeneration in the amygdala in representative cases.
(A-F) Pathological findings in a case with Braak NFT stage II, Thal phase 0, amygdala GFA stage 2, Saito AG stage III, and LATE-NC stage 2.
(A, B) Phosphorylated TDP-43 accumulation in the amygdala (A) and dentate gyrus in the hippocampus (B). pS409/410 immunohistochemistry. Scale bar: 25 μm.
(C) A GFA in the amygdala. AT8 immunohistochemistry. Scale bar: 25 μm.
(D) AGs in the amygdala. Gallyas method. Scale bar: 25 μm.
(E) Hippocampal sclerosis. Hematoxylin-eosin stain. Scale bar: 100 μm.
(F) Severe loss of neurons with gliosis in the amygdala. Hematoxylin-eosin stain. Scale bar: 25 μm.
(G-L) Pathological findings in a case with Braak stage II, Thal phase 0, amygdala GFA stage 4, Saito AG stage III, and LATE-NC stage 0.
(G) This case lacked phosphorylated TDP-43-positive lesion in any region. The amygdala. pS409/410 immunohistochemistry. Scale bar: 25 μm.
(H, I) GFAs in the amygdala. AT8 immunohistochemistry. Scale bar: 25 μm.
(J) AGs in the amygdala. Gallyas method. Scale bar: 25 μm.
(K) Neither loss of pyramidal neurons nor gliosis is noted in the hippocampal CA1. Hematoxylin-eosin stain. Scale bar: 100 μm.
(L) Severe neuronal loss with gliosis in the amygdala. Hematoxylin-eosin stain. Scale bar: 25 μm.
